# Prevention of antipsychotic-induced hyperglycaemia by vitamin D: a data mining prediction followed by experimental exploration of the molecular mechanism

**DOI:** 10.1038/srep26375

**Published:** 2016-05-20

**Authors:** Takuya Nagashima, Hisashi Shirakawa, Takayuki Nakagawa, Shuji Kaneko

**Affiliations:** 1 Department of Molecular Pharmacology, Graduate School of Pharmaceutical Sciences, Kyoto University, 46-29 Yoshida-Shimoadachi-cho, Sakyo-ku, Kyoto 606-8501, Japan; 2 Department of Clinical Pharmacology and Therapeutics, Kyoto University Hospital, 54 Shogoin-Kawahara-cho, Sakyo-ku, Kyoto 606-8507, Japan

## Abstract

Atypical antipsychotics are associated with an increased risk of hyperglycaemia, thus limiting their clinical use. This study focused on finding the molecular mechanism underlying antipsychotic-induced hyperglycaemia. First, we searched for drug combinations in the FDA Adverse Event Reporting System (FAERS) database wherein a coexisting drug reduced the hyperglycaemia risk of atypical antipsychotics, and found that a combination with vitamin D analogues significantly decreased the occurrence of quetiapine–induced adverse events relating diabetes mellitus in FAERS. Experimental validation using mice revealed that quetiapine acutely caused insulin resistance, which was mitigated by dietary supplementation with cholecalciferol. Further database analysis of the relevant signalling pathway and gene expression predicted quetiapine-induced downregulation of *Pik3r1*, a critical gene acting downstream of insulin receptor. Focusing on the phosphatidylinositol 3-kinase (PI3K) signalling pathway, we found that the reduced expression of *Pik3r1* mRNA was reversed by cholecalciferol supplementation in skeletal muscle, and that insulin-stimulated glucose uptake into C2C12 myotube was inhibited in the presence of quetiapine, which was reversed by concomitant calcitriol in a PI3K-dependent manner. Taken together, these results suggest that vitamin D coadministration prevents antipsychotic-induced hyperglycaemia and insulin resistance by upregulation of PI3K function.

Atypical antipsychotics such as quetiapine and olanzapine are clinically used to treat a wide variety of mental disorders, including schizophrenia, bipolar disorder, depression and sleep disorders. However, these drugs are often associated with the occurrence of hyperglycaemia and other adverse events, thus limiting their clinical use^1^,^2^. Antipsychotic-induced hyperglycaemia frequently leads to new-onset diabetes mellitus (DM), occasionally results in life-threatening diabetic ketoacidosis and coma, and, in the worst-case scenario, culminates in death^3^.

Despite wide recognition of the relationship between atypical antipsychotics and hyperglycaemia/DM, the pathogenesis of drug-induced DM remains poorly understood. Proposed mechanisms for antipsychotic-induced DM include weight gain, decreased insulin secretion from pancreatic β-cells, insulin resistance and impaired leptin action^4^. Although some clinical studies show that the antidiabetic agent, metformin, is effective against antipsychotic-associated DM, additional treatments are required because antipsychotic-induced hyperglycaemia is multifactorial, which makes it difficult to treat with a single-drug therapy^5^.

The FDA Adverse Event Reporting System (FAERS) is the largest worldwide database of the self-reports of adverse drug events, and freely available to the public. Several researchers have successfully identified previously unknown drug-drug interactions through analysis of FAERS^6^,^7^,^8^. In particular, Zhao and colleagues developed a FAERS-based approach to identify a concomitant drug B that might mitigate the risk of adverse events associated with the use of a drug A^8^. Here, we first explored the FAERS database to determine an effective drug combination to lower the occurrence of antipsychotic-induced hyperglycaemia, and then ascertained the validity of the prospective drug combination in a mouse model of antipsychotic-induced glucose intolerance. We also predicted the molecular mechanism of antipsychotic-induced hyperglycaemia by combining metabolic pathway data from the Kyoto Encyclopaedia of Genes and Genomes (KEGG) PATHWAY database^9^,^10^ with a comprehensive rat toxicogenomics database, DrugMatrix. The DrugMatrix database contains microarray gene expression profiles^11^ deposited in the Gene Expression Omnibus (GEO) of the United States National Centre for Biotechnology Information^12^,^13^. Experimentally, we performed a reverse transcription-polymerase chain reaction (RT-PCR) analysis to confirm the predicted changes in gene expression in murine skeletal muscle. We further validated the hypothesis by *in vitro* experiments using C2C12 mouse myoblast cell line. The results of the current data mining study with experimental validation suggest that vitamin D can prevent quetiapine-induced hyperglycaemia through inhibition of insulin resistance via the upregulation of phosphatidylinositol 3-kinase (PI3K) function.

## Results

### Atypical antipsychotics increase the risk of DM-related adverse events in the FAERS database

We first investigated the association between atypical antipsychotic usage and DM-related adverse events in the FAERS database (Table 1). The search terms for DM-related adverse events are described in Supplementary Table S1. A disproportionality analysis of the database revealed associations between antipsychotic use and the increased occurrence of DM-related events. A strong association (odds ratio (OR) >20) was observed for quetiapine and olanzapine, and an intermediate association (5 < OR < 20) was observed for risperidone, aripiprazole and ziprasidone. Meanwhile, the association for clozapine was low (OR <5). These findings confirm the hypothesis that the risk of atypical antipsychotic-induced hyperglycaemia is properly reflected in database search. In the following analysis, we focused on quetiapine due to the high OR and the large number of DM reports linked to quetiapine usage.

### Quetiapine-induced DM is observed regardless of confounding variables

We next investigated the involvement of confounding variables in quetiapine-induced, DM-related adverse events (Table 2). The association between quetiapine use and the increased risk of DM was observed even when each major adverse event search term (e.g., “diabetes mellitus”, “diabetic ketoacidosis”, “diabetic coma”) was entered separately into the analysis. When affected individuals were stratified by age, the OR was greatest in the 40–49-year-old group, followed by the <40-year-old group, 50–59-year-old group, 60–69-year-old group and >70-year-old group, consistently with a previous pharmacoepidemiological study^2^. When individuals were stratified by gender, the increased occurrence of DM was observed in both male and female patients, with almost equal ORs. After restricting the patient population to those exhibiting specific primary diseases (e.g., schizophrenia or bipolar disorder), the DM frequency was still highly significant. Likewise, the increased incidence of DM was observed independently of concomitant psycholeptic agents, such as haloperidol or lithium. These results suggest that quetiapine use is significantly associated with an increased occurrence of DM-related adverse events, regardless of age, gender, primary diseases or combinatorial drug therapy.

### Vitamin D decreases the occurrence of atypical antipsychotic-induced, DM-related adverse events

We next searched for coexisting drugs with the capacity to decrease the risk of quetiapine-induced DM, irrespective of their molecular mechanisms of action (Table 3). Consequently, pregabalin, lamotrigine and vitamin D analogues (see Supplementary Table S2 for search terms for vitamin D analogues) were associated with a decreased occurrence of quetiapine-induced, DM-related adverse events. The lowest OR was observed for vitamin D. The mitigating effect of vitamin D against DM was also observed in combinatorial therapy with olanzapine (OR = 0.21, 95% confidence interval (CI) = 0.14–0.32). Vitamin D itself did not affect the frequency of DM-related adverse events (OR = 1.01, 95% CI = 0.94–1.07, *p* = 0.86), implying that vitamin D may interact with atypical antipsychotics to lessen the risk of hyperglycaemia. Below, we focused on vitamin D rather than the other candidate drugs, because pregabalin (OR = 1.33, 95% CI = 1.25–1.41) or lamotrigine (OR = 1.41, 95% CI = 1.30–1.52) was in itself associated with a slightly increased risk of DM-related adverse events.

### Cholecalciferol alleviates quetiapine-induced hyperglycaemia and hyperinsulinemia in mice

**Figure 1:**
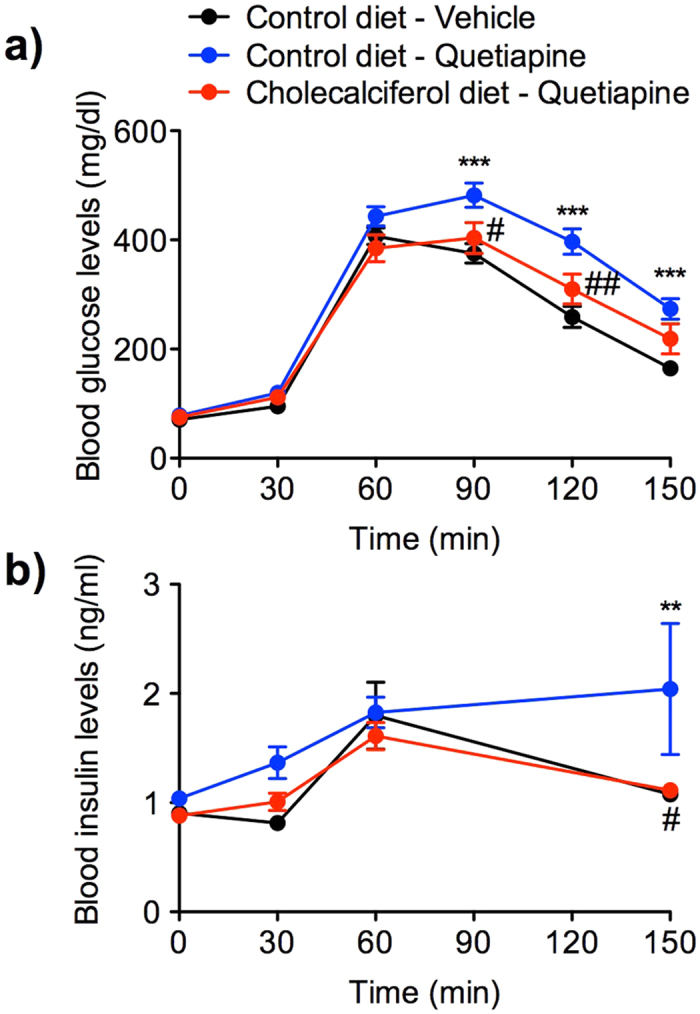
Effects of dietary vitamin D/cholecalciferol supplementation on quetiapine-induced increases in blood glucose and insulin levels. Mice were fed a control or cholecalciferol-supplemented diet (1200 IU cholecalciferol/day) for 1 week, fasted for 16 h, and given quetiapine (10 mg/kg, i.p.) or vehicle 30 min before injection of d-glucose (3 g/kg, i.p.). (**a**) Blood glucose (*n* = 14) and (**b**) blood insulin (*n* = 8) levels were measured at the indicated times. Data are given as means ± standard error of the mean (SEM; **p < 0.01, ***p < 0.001 vs. vehicle-treated group; ^#^*p* < 0.05, ^##^*p* < 0.01 vs. quetiapine-treated group).

To verify the prediction that vitamin D mitigates the risk of quetiapine-induced hyperglycaemia, we assessed the effect of the agent against quetiapine-induced glucose intolerance in an animal model (Fig. 1). Male ICR mice were fed a control or vitamin D (cholecalciferol)-supplemented diet (1200 IU cholecalciferol/day) for 1 week, fasted for 16 h and subjected to an intraperitoneal glucose tolerance test. Mouse body weights were unaffected by cholecalciferol diet (Supplementary Fig. S1). Administration of quetiapine (10 mg/kg, i.p.) to mice receiving the control diet acutely led to significantly higher levels of glucose (two-way analysis of variance (ANOVA): *F*_1,130_ = 24.32, *p* < 0.001) and insulin (two-way ANOVA: *F*_1,42_ = 4.74, *p* < 0.05) in the blood. On the other hand, dietary cholecalciferol supplementation significantly prevented the quetiapine-induced increases in both blood glucose and insulin (two-way ANOVA: *F*_1,130_ = 6.44, *p* < 0.05; *F*_1,42_ = 4.81, *p* < 0.05, respectively). The cholecalciferol diet did not modify the anti-psychostimulant effect of quetiapine on methamphetamine-promoted hyperlocomotion (Supplementary Fig. S2). Because hyperinsulinemia is a main feature of the insulin-resistant state^14^, our findings suggest that vitamin D averts quetiapine-induced hyperglycaemia by defending against insulin resistance.

### Database search for signalling pathways and gene expression changes potentially responsible for quetiapine-induced insulin resistance

**Figure 2:**
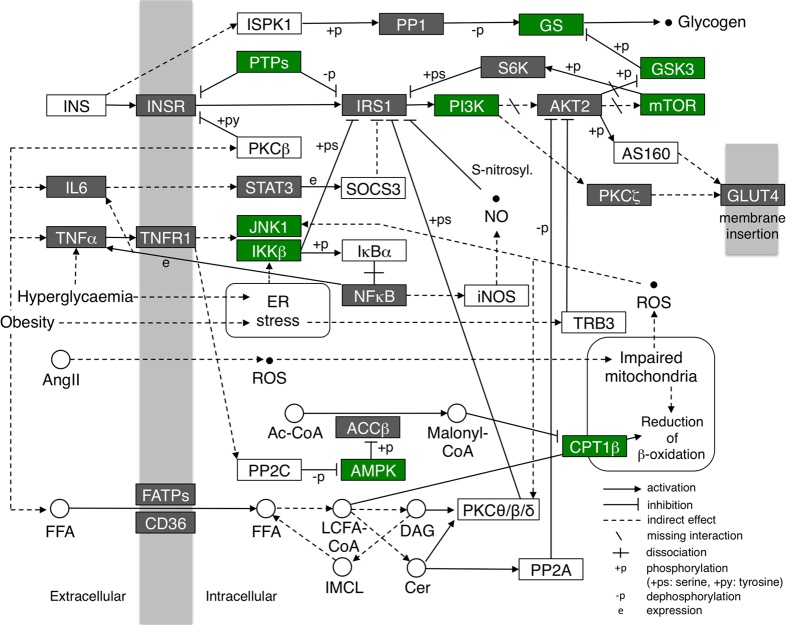
Candidate genes for action mechanism of quetiapine-induced insulin resistance, as predicted by combined analysis of GEO and KEGG PATHWAY databases. The insulin resistance pathway map in skeletal muscle cells was derived from the KEGG PATHWAY database (Entry No. map04931), with slight modifications. Changes in gene expression after oral quetiapine administration (500 mg/kg, 5 days of exposure with daily dosing) were investigated using GEO microarray data (GEO Accession No. GSE59923). Genes with expression levels measured in the microarray data are shown in green (significantly altered by quetiapine administration) or grey (not significantly altered by quetiapine administration). Ac-CoA = acetyl-coenzyme A. ACCβ = acetyl-coenzyme A carboxylase β. AMPK = AMP-activated protein kinase. AngII = angiotensin II. AS160 = Akt substrate of 160 kDa. Cer = ceramide. CPT1β = carnitine palmitoyltransferase 1 β. DAG = diacylglycerol. FATPs = fatty acid transport proteins. FFA = free fatty acid. GLUT4 = glucose transporter type 4. GS = glycogen synthase. GSK3 = glycogen synthase kinase 3. IKKβ = inhibitor of nuclear factor-kappa-B kinase subunit β. IL6 = interleukin 6. IMCL = intramyocellular lipid. iNOS = inducible nitric oxide synthase. INS = insulin. INSR = insulin receptor. IRS1 = insulin receptor substrate 1. ISPK1 = insulin-stimulated protein kinase 1. IκBα = nuclear factor-kappa-B inhibitor α. JNK1 = c-Jun N-terminal kinase 1. LCFA-CoA = long-chain fatty acyl-coenzyme A. mTOR = mechanistic target of rapamycin. NFκB = nuclear factor-κ-B. NO = nitric oxide. PI3K = phosphatidylinositol 3-kinase. PKC = protein kinase C. PP = protein phosphatase. PTPs = protein tyrosine phosphatases. ROS = reactive oxygen species. S6K = p70 ribosomal S6 kinase. SOCS3 = suppressor of cytokine signalling 3. STAT3 = signal transducer and activator of transcription 3. TNFR1 = tumor necrosis factor receptor 1. TNFα = tumor necrosis factor α. TRB3 = tribbles homolog 3.

To clarify the molecular mechanisms underlying quetiapine-induced hyperglycaemia, we investigated toxicogenomics microarray datasets published in GEO (Accession No. GSE59923) by focusing on the expression of 24 gene products included in the KEGG “insulin resistance” pathway (Entry No. map04931; Fig. 2). In GEO, the expression levels of ten insulin resistance-related genes were downregulated in rat liver after oral quetiapine administration (500 mg/kg, 5 days of exposure with daily dosing; Table 4), while only one gene was upregulated. The ten downregulated genes encode nine relevant proteins downstream of insulin receptors, glycogen synthesis and tumour necrosis factor receptors. Among them, the most prominent reduction in expression was observed for *Pik3r1*, which encodes a known risk factor for DM, phosphatidylinositol 3-kinase (PI3K)^15^,^16^. Therefore, we speculated that vitamin D lessens quetiapine-induced hyperglycaemia by inhibiting the downregulation of *Pik3r1*/PI3K and subsequent induction of insulin resistance.

### Cholecalciferol prevents quetiapine-induced downregulation of *Pik3r1 in vivo*

**Figure 3:**
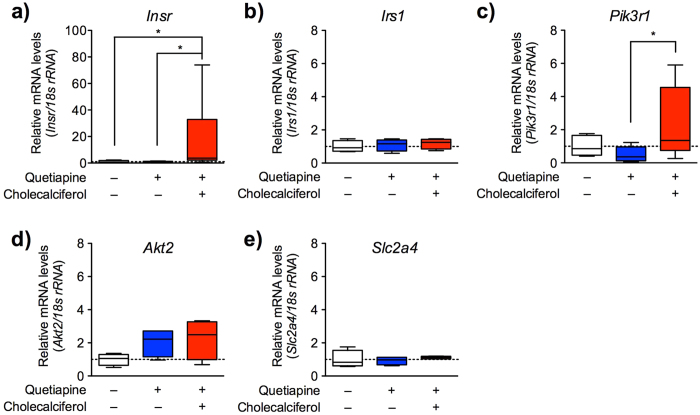
Effects of quetiapine and vitamin D/cholecalciferol supplementation on the expression levels of genes related to the insulin receptor signalling pathway. Mice were fed a control or cholecalciferol-supplemented diet and given quetiapine (10 mg/kg, i.p.) or vehicle, as described in Fig. 1. Immediately after a 150 min glucose tolerance test, the right thigh muscles were collected, and the gene expression levels of *Insr* (**a**), *Irs1* (**b**), *Pik3r1* (**c**), *Akt2* (**d**) and *Slc2a4* (**e**) were analysed by quantitative real-time RT-PCR. Each expression level was normalised to that of *18s rRNA* and presented relative to the group receiving the control diet plus vehicle. Data are shown as box-whisker plots, where the horizontal lines indicate the medians, the boxes indicate the 25th to 75th percentiles and the whiskers indicate the entire range (*n* = 4–8; *p < 0.05).

We next confirmed the prediction that vitamin D can prevent quetiapine-induced insulin resistance in a mouse model by examining the ability of cholecalciferol diet to block drug-provoked changes in gene expression related to the insulin receptor signalling pathway (Fig. 3). Immediately after administration of the glucose tolerance test in mice, mRNA levels were measured via quantitative RT-PCR in skeletal muscle, the predominant site of insulin-mediated glucose uptake^17^. Although not statistically significant (Welch’s *t*-test: *p* = 0.079), downregulation of *Pik3r1* mRNA was observed even at 150 min after treatment with quetiapine. However, the quetiapine-facilitated downregulation of *Pik3r1* was significantly reversed by dietary cholecalciferol supplementation (Kruskal-Wallis test value = 6.135, *p* < 0.05; Dunn’s test, *p* < 0.05). Significant upregulation of *Insr* (encoding the insulin receptor) was also observed in the cholecalciferol group (Kruskal-Wallis test value = 10.55, *p* < 0.01), consistently with a previous report^18^. No significant change was found in the expression levels of other relevant genes, including *Irs1* (encoding insulin receptor substrate 1; Kruskal-Wallis test value = 0.6154, *p* = 0.7351), *Akt2* (encoding thymoma viral proto-oncogene 2; Kruskal-Wallis test value = 3.038, *p* = 0.2189) or *Slc2a4* (encoding solute carrier family 2 (facilitated glucose transporter), member 4; Kruskal-Wallis test value = 1.885, *p* = 0.3897). These experimental data indicate that vitamin D counteracts quetiapine-induced insulin resistance by blocking *Pik3r1* downregulation, validating the prediction obtained from analysis of the GEO and KEGG PATHWAY databases.

### Calcitriol prevents quetiapine-induced insulin resistance via PI3K signalling pathway *in vitro*

**Figure 4:**
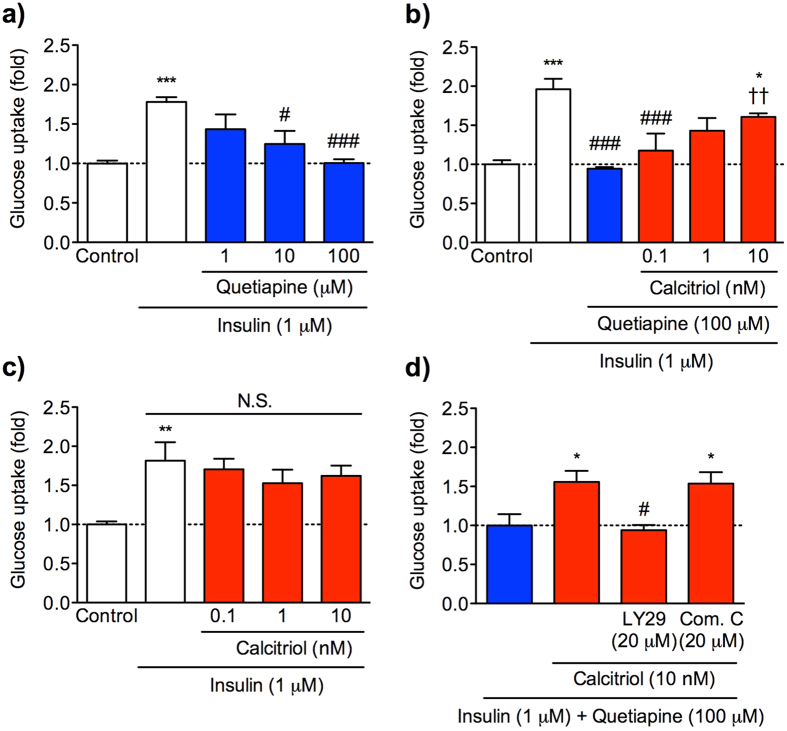
Calcitriol improved quetiapine-induced insulin resistance through PI3K signalling pathway in C2C12 myotubes. Glucose uptake was evaluated using 2-NBDG as described in Materials and Methods. (**a**) Concentration-dependent effect of quetiapine on insulin-stimulated glucose uptake. Differentiated C2C12 cells were treated with quetiapine (1–100 μM) for 1 h. The cells were then stimulated with or without insulin (1 μM) for 15 min. *n* = 10. ***p < 0.001 vs. control group; ^#^*p* < 0.05, ^###^*p* < 0.001 vs. insulin-treated group. (**b**) Concentration-dependent effect of calcitriol on quetiapine-induced insulin resistance. Differentiated C2C12 cells were treated with calcitriol (0.1–10 nM) for 24 h, followed by quetiapine (100 μM) for 1 h. The cells were then stimulated with or without insulin (1 μM) for 15 min. *n* = 10. *p < 0.05, ***p < 0.001 vs. control group; ^###^*p* < 0.001 vs. insulin-treated group; ^††^*p* < 0.01 vs. quetiapine-treated group. (**c**) Null effect of calcitriol on insulin-stimulated glucose uptake. Differentiated C2C12 cells were treated with calcitriol (0.1–10 nM) for 24 h. The cells were then stimulated with or without insulin (1 μM) for 15 min. *n* = 9. **p < 0.01 vs. control group; N.S. = not significant. (**d**) Effect of LY294002 (PI3K inhibitor) or compound C (AMPK inhibitor) on the improvement of quetiapine-induced insulin resistance by calcitriol. Differentiated C2C12 cells were treated with calcitriol (10 nM) for 24 h, followed by quetiapine (100 μM) in the presence or absence of LY294002 (LY29; 20 μM) or compound C (Com. C; 20 μM) for 1 h. The cells were then stimulated with insulin (1 μM) for 15 min. *n* = 9–13, *p < 0.05 vs. quetiapine-treated group; ^#^*p* < 0.05 vs. quetiapine and calcitriol-treated group. Data are given as means ± SEM relative to the control (**a–c**) or insulin and quetiapine-treated (**d**) group.

We finally validated the hypothesis by glucose uptake assays using C2C12 mouse myotubes (Fig. 4). Treatment with quetiapine (1–100 μM) for 1 h inhibited the insulin-stimulated glucose uptake in a concentration-dependent manner. Significant inhibition was observed at 10 and 100 μM quetiapine (Fig. 4a), suggesting the induction of insulin resistance. The insulin resistance induced by quetiapine (100 μM) was improved by pretreatment with calcitriol (0.1–10 nM; 1,25-dihydroxycholecalciferol, the biologically active form of cholecalciferol/vitamin D_3_) for 24 h in a concentration-dependent manner, and significant improvement was achieved at 10 nM calcitriol (Fig. 4b). Calcitriol pretreatment itself had no effect on the insulin-stimulated glucose uptake at any concentrations examined (Fig. 4c). The improvement of quetiapine-induced insulin resistance by calcitriol was still observed when co-treated with an AMP-activated protein kinase (AMPK) inhibitor compound C (20 μM), but was significantly blocked in the presence of a PI3K inhibitor LY294002 (20 μM, Fig. 4d). These data demonstrate that vitamin D improves quetiapine-induced insulin resistance via stimulating PI3K signalling pathway.

## Discussion

The current data mining predictive analysis with experimental validation provides the first indication that vitamin D can prevent antipsychotic-induced hyperglycaemia by suppressing insulin resistance via upregulation of *Pik3r1*.

Little is known about the impact of vitamin D on metabolic abnormalities caused by atypical antipsychotics. Two pilot clinical studies assessed the efficacy of vitamin D against antipsychotic-induced metabolic side effects, but both were limited by a statistically insufficient sample size^19^,^20^. Another investigation suggested the involvement of vitamin D deficiency in antipsychotic-induced hyperglycaemia^21^. However, this study drew its conclusion from animal models whose weight gain was suppressed by clozapine treatment, which is inconsistent with clinical observations. By contrast, our results are supported by the analysis of human clinical databases as well as pharmacological experiments both *in vivo* and *in vitro*, thus providing strong evidence and a molecular basis for the efficacy of vitamin D as a preventative treatment against antipsychotic-induced hyperglycaemia. Our findings are also in line with a previous report showing that vitamin D may prevent the development of type 2 DM^22^.

The North American Association for the Study of Obesity reported an increased risk of DM for clozapine and olanzapine, an inconsistent risk for quetiapine and risperidone, and no risk for aripiprazole and ziprasidone^1^. Nevertheless, our FAERS analysis indicated that clozapine is associated with a relatively low risk of DM. This discrepancy might be explained by a lower number of adverse event reports for clozapine-induced DM in the FAERS database, possibly because glucose dysregulation following clozapine use is easily detected and rapidly managed via mandatory blood monitoring^23^. On the other hand, a relatively higher DM risk was found for aripiprazole and ziprasidone in FAERS, perhaps due to antipsychotic polypharmacy. In fact, our additional analysis of FAERS showed that nearly 85% (1368/1613) of DM patients who received ziprasidone also received quetiapine. The primary suspect drug for DM was quetiapine in 77.5% of these cases (1250/1613), as opposed to ziprasidone in only 5.6% of the cases (91/1613).

Vitamin D mediates biological responses by binding to nuclear vitamin D receptors that function as transcription factors to regulate target gene expression^24^. Here, we found that dietary vitamin D supplementation upregulated *Pik3r1* in an animal model of antipsychotic-induced glucose intolerance. In addition, the improvement of antipsychotic-induced insulin resistance by calcitriol was blocked in the presence of PI3K inhibitor in our *in vitro* experiments, further supporting the functional involvement of *Pik3r1*. Mice lacking both *Pik3r1* and *Pik3r2* in the skeletal muscles exhibit severely impaired PI3K signalling and insulin resistance^25^. Akt is the major downstream effector of PI3K and promotes the membrane translocation of glucose transporter 4 (GLUT4), thus increasing cellular glucose uptake^26^. Altogether, these findings suggest that *Pik3r1* downregulation promotes hyperglycaemia by impairing GLUT4-facilitated glucose uptake. Meanwhile, the upregulation of *Insr* by dietary vitamin D supplementation may help to overturn glucose intolerance.

Nonetheless, our data do not preclude other possible mechanisms of antipsychotic/vitamin D action. For example, atypical antipsychotics and vitamin D both reportedly affect insulin resistance by regulating the phosphorylation of insulin signalling pathway proteins (e.g., IRS1 and Akt) in a non-genomic manner^27^,^28^,^29^. Moreover, antipsychotics decrease insulin secretion from pancreatic β-cells by promoting cellular apoptosis^30^,^31^, while vitamin D improves pancreatic β-cell function^22^. Further investigation is required to determine to what extent the expression of *Pik3r1* and insulin resistance are affected by a long-term treatment with quetiapine and/or vitamin D.

Databases like FAERS and GEO are useful tools for investigating drug toxicity, but data mining analyses are fraught with many limitations in terms of generating hypotheses. For instance, we could not obtain the preventive effect of quetiapine-induced DM for metformin by the analysis of FAERS (OR = 5.29, 95% CI = 4.90–5.71) because of the indication bias. In addition, even though a large number of toxicogenomics datasets are registered in GEO, the comprehensiveness of reported experimental conditions is presently insufficient. However, these shortcomings can be overcome by integration with systems pharmacology databases^32^ or by meta-analysis of large-scale toxicogenomic data^33^. Despite the challenges for prediction accuracy, data mining is still useful in areas where traditional clinical investigations and animal experiments fail to provide insights into molecular mechanisms of complicated conditions, such as antipsychotic-induced hyperglycaemia.

In conclusion, this study demonstrated the efficacy of vitamin D against antipsychotic-induced hyperglycaemia using data mining prediction followed by experimental validation both *in vivo* and *in vitro*. Based on the current results, we propose a novel vitamin D/antipsychotic combination pharmacotherapy in which vitamin D can efficaciously safeguard against antipsychotic-induced hyperglycaemia accompanied by insulin resistance. Thus, the analysis of rapidly growing databases of clinical adverse events and molecular toxicity can provide insights into the practical management of undesirable adverse drug events, as well as their molecular mechanisms.

## Materials and Methods

### Analysis of the FAERS database

FAERS adverse event reports were obtained from the FDA website (http://www.fda.gov/Drugs/GuidanceComplianceRegulatoryInformation/Surveillance/AdverseDrugEffects/). Duplicated reports (among a total of 5,821,354 reports) from the first quarter of 2004 through the second quarter of 2014 were filtered by applying the FDA’s recommendation of adopting the most recent case number. Consequently, 4,547,841 remaining reports were analysed in this study. Arbitrary drug names, including trade names and abbreviations, were mapped into unified generic names via text mining. Adverse event risk was evaluated by calculating the reporting ORs with a 95% CI according to methods described in the literature^34^.

Briefly, individuals in the FAERS database were divided into the following four groups: (a) individuals who received the drug of interest (i.e., quetiapine or vitamin D) and exhibited DM-related adverse events; (b) individuals who received the drug of interest, but did not exhibit DM-related adverse events; (c) individuals who did not receive the drug of interest and exhibited DM-related adverse events; and (d) individuals who did not receive the drug of interest and did not exhibit DM-related adverse events. The OR with 95% CI was defined as follows:


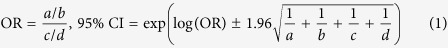


where a, b, c and d refer to the number of individuals in each group, and log refers to the natural logarithm. If the lower limit of the 95% CI was >1, a significant association was assumed between use of the drug of interest (i.e., quetiapine) and the increased occurrence of DM-related adverse events. Contrarily, if the upper limit of the 95% CI was <1, a significant association was assumed between use of the drug of interest (i.e., vitamin D) and the decreased occurrence of DM-related adverse events.

The search terms for “DM” and “vitamin D” are described in Supplementary Tables 1 and 2, respectively. We regarded drug indication data as the patients’ primary diseases. To search for concomitant drugs associated with a decreased occurrence of quetiapine-induced, DM-related adverse events, the number of individuals treated with the concomitant drug of interest was restricted to a minimum of 1000, because ORs are prone to fluctuation when the sample size is small.

### Combinatorial analysis of the GEO and KEGG PATHWAY databases

Microarray gene expression profiles were obtained from a previous study published in the GEO database (GEO Accession No. GSE 59923; http://www.ncbi.nlm.nih.gov/geo/query/acc.cgi? acc=GSE59923). Expression data were analysed in the livers of rats orally treated with quetiapine (500 mg/kg, 5 days of exposure with daily dosing) or vehicle (carboxymethylcellulose). The analysis included genes participating in the insulin resistance pathway in skeletal muscle cells, and obtained from the KEGG PATHWAY database (Entry No. map04931; http://www.kegg.jp/dbget-bin/www_bget? pathway+map04931). For the expression analysis of each gene, log2-transformed microarray data were back-transformed to the original scale and presented relative to the vehicle-treated group.

### Animals

All animal experiments were approved by the Kyoto University Animal Research Committee in accordance with the ethical guidelines of the Committee. All experiments were designed to minimise the use of animals and number of experiments. Male ICR mice (5–6 weeks of age) were purchased from Japan SLC (Shizuoka, Japan) and housed with a constant ambient temperature (24 ± 1 °C) and humidity (55% ± 10%) on a 12 h/12 h light/dark cycle. Food and water were freely available, except for during the fasting period before the glucose tolerance test.

### Drugs and reagents

Quetiapine was purchased from Wako Pure Chemical Industries (Osaka, Japan). Methamphetamine was from Sumitomo Dainippon Pharma (Osaka, Japan). d-Glucose was from Nacalai Tesque (Kyoto, Japan). Insulin was from Biological Industries (Cromwell, CT, USA). 2-Deoxy-2-[(7-nitro-2,1,3-benzoxadiazol-4-yl)amino]-d-glucose (2-NBDG), LY294002, and dorsomorphin (compound C) were from Cayman Chemical (Ann Arbor, MI, USA). Calcitriol was from Toronto Research Chemicals (Ontario, Canada). Dulbecco’s modified Eagle’s medium (DMEM) and foetal bovine serum (FBS) were from Sigma-Aldrich (Saint-Louis, MO, USA). Horse serum (HS) was from Invitrogen Japan (Tokyo, Japan).

For *in vivo* study, quetiapine and methamphetamine were dissolved in distilled water plus 1% Tween 80 prior to use. d-Glucose was dissolved in distilled water before use. Quetiapine, methamphetamine and d-glucose were administered intraperitoneally at a volume of 10 ml/kg body weight.

Vitamin D_3_ (cholecalciferol) was administered orally in the diet. MF diet (Oriental Yeast, Tokyo, Japan) containing 1370 IU vitamin D_3_/kg (8 IU vitamin D_3_/day based on daily consumption of 6 g chow/30 g body weight) served as the control diet. The cholecalciferol-supplemented diet consisted of a mixture of MF diet and 200,000 IU vitamin D_3_/kg (1200 IU/day).

### Intraperitoneal glucose tolerance test

Mice were fed a control or cholecalciferol-supplemented diet for 1 week, followed by a 16 h fast before a 150 min intraperitoneal glucose tolerance test. Quetiapine (10 mg/kg) or vehicle (1% Tween 80) was administered intraperitoneally before the injection of d-glucose (3 g/kg). Blood samples were collected from the tail vein at the indicated time points, and blood glucose levels were measured using an Accu-Chek Blood Glucose Meter (Roche Diagnostics, Almere, the Netherlands). Blood insulin levels were measured using a Mouse Insulin Enzyme-Linked Immunosorbent Assay Kit (Morinaga Institute of Biological Science, Yokohama, Japan) and 5 μl blood samples collected at the indicated time points, as instructed by the manufacturer.

### Real-time quantitative RT-PCR

Immediately after the glucose tolerance test, mice were sacrificed by cervical dislocation, and the right thigh muscles were collected. Total RNA was isolated using the ISOGEN Reagent (Nippon Gene, Tokyo, Japan), and the isolated RNA (0.5 μg) was reverse transcribed using the ReverTra Ace qPCR RT Kit (Toyobo, Osaka, Japan). Real-time quantitative RT-PCR was performed using the StepOne Real-Time PCR System (Life Technologies, Carlsbad, CA, USA) and the THUNDERBIRD SYBR qPCR Mix (Toyobo). Each PCR amplification consisted of heat activation for 10 min at 95 °C, followed by 40 cycles at 95 °C for 15 s and 60 °C for 1 min.

The oligonucleotide primers used for RT-PCR were as follows: 5′-GTA ACC CGT TGA ACC CCA TT-3′ and 5′-CCA TCC AAT CGG TAG TAG CG-3′ for the 18s ribosomal RNA gene (*18s rRNA*); 5′-CCC AGG CCA TCC CGA AAG-3′ and 5′-TCT CAA ATG GCC TGT GCT CC-3′ for *Insr*; 5′-TTA GGC AGC AAT GAG GGC AA-3′ and 5′-TCT TCA TTC TGC TGT GAT GTC CA-3′ for *Irs1*; 5′-GAC AGC GAA GCG ACG GC-3′ and 5′-GTC TGA TTT TAC TGC CAC GCT C-3′ for *Pik3r1*; 5′-CGC TTG CGG TCT GAT GTT TT-3′ and 5′-AAT ACC GCC TTT TCC AGC CA-3′ for *Akt2*; and 5′-TTA TTG CAG CGC CTG AGT CT-3′ and 5′-GGG TTC CCC ATC GTC AGA G-3′ for *Slc2a4*. The mRNA expression levels of each gene were normalised to that of *18s rRNA*, which was measured in parallel in each sample and expressed relative to the vehicle-treated group.

### Cell culture and differentiation

Mouse C2C12 myoblast cell line was kindly provided by Prof. H. Takeshima (Kyoto University, Graduate School of Pharmaceutical Sciences, Kyoto, Japan). The cells were cultured in 100-mm dishes in DMEM containing 10% heat-inactivated FBS at 37 °C with 5% CO_2_. One day after seeding cells in black 96-well plates (>70% confluence), the medium was switched to DMEM with 2% HS to differentiate cells into myotubes. The myotubes were used for experiments 3–5 days following differentiation.

### Glucose uptake assay

Glucose uptake assay was performed by measuring the uptake of 2-NBDG, a fluorescent derivative of glucose. Differentiated myotubes were starved in a serum-free DMEM for 3 h before treatment with the drugs. After treatment, cells were stimulated with or without insulin (1 μM) dissolved in KRPH buffer (136 mM NaCl, 4.7 mM KCl, 1 mM MgSO_4_, 1 mM CaCl_2_, 5 mM KH_2_PO_4_, 20 mM HEPES) for 15 min followed by the addition of 2-NBDG (50 μM) for 20 min. After incubation, free 2-NBDG was washed out 3 times with KRPH buffer. The fluorescence retained in the cells was measured with a fluorescence microplate reader (FDSS/μCell; Hamamatsu Photonics, Shizuoka, Japan) at an excitation wavelength of 480 nm and an emission wavelength of 540 nm.

### Statistical analysis

Statistical analyses of the FAERS and GEO databases were performed with R version 3.1.2 Software (R Foundation for Statistical Computing, Vienna, Austria). Data obtained from the animal experiments were analysed with GraphPad Prism 5 Software (GraphPad, San Diego, CA, USA). Differences between two groups were compared via an unpaired two-tailed *t*-test with Welch’s correction, while differences between more than two groups were compared via the Kruskal-Wallis test followed by Dunn’s post hoc test (Fig. 3) or the one-way ANOVA followed by Tukey’s post hoc test (Fig. 4). Time-course data were analysed by applying a two-way ANOVA for repeated measures, followed by the Bonferroni post hoc test. Differences were considered significant at *p* < 0.05.

## Additional Information

**How to cite this article**: Nagashima, T. *et al*. Prevention of antipsychotic-induced hyperglycaemia by vitamin D: a data mining prediction followed by experimental exploration of the molecular mechanism. *Sci. Rep.* **6**, 26375; doi: 10.1038/srep26375 (2016).

## Supplementary Material

Supplementary Information

## Figures and Tables

**Table 1:** Association between atypical antipsychotics (drug A) and the occurrence of DM in FAERS.

**Drug A**	**DM without drug A (%)**	**DM with drug A (%)**	**Odds ratio (95% CI)**
Quetiapine	37373/4482388 (0.83)	10809/65453 (16.51)	23.53 (22.99–24.07)
Olanzapine	41668/4515358 (0.92)	6514/32483 (20.05)	26.93 (26.17–27.72)
Risperidone	44382/4513579 (0.98)	3800/34262 (11.09)	12.56 (12.13–13.01)
Aripiprazole	46007/4518947 (1.02)	2175/28894 (7.53)	7.91 (7.57–8.28)
Ziprasidone	46569/4534867 (1.03)	1613/12974 (12.43)	13.68 (12.98–14.43)
Clozapine	47124/4517820 (1.04)	1058/30021 (3.52)	3.47 (3.26–3.69)

Representative atypical antipsychotics were compared. The search terms for “DM” (including related adverse events) are described in Supplementary Table S1.

**Table 2:** Potential confounding variables for the quetiapine-induced DM in FAERS.

**Group**	**DM without quetiapine (%)**	**DM with quetiapine (%)**	**Odds ratio (95% CI)**
Limited by an adverse event name			
Diabetes mellitus	20092/4482388 (0.45)	7668/65453 (11.72)	29.47 (28.67–30.30)
Diabetic ketoacidosis	2897/4482388 (0.06)	1258/65453 (1.92)	30.30 (28.35–32.39)
Diabetic coma	885/4482388 (0.02)	876/65453 (1.34)	68.69 (62.55–75.44)
Stratified by age			
<40 years	3622/657664 (0.55)	2229/16317 (13.66)	28.57 (27.03–30.20)
40–49 years	3982/391629 (1.02)	2813/11828 (23.78)	30.38 (28.82–32.02)
50–59 years	5929/547124 (1.08)	1690/10574 (15.98)	17.36 (16.39–18.40)
60–69 years	5170/536481 (0.96)	409/4932 (8.29)	9.29 (8.37–10.32)
≥70 years	4100/616472 (0.67)	175/5067 (3.45)	5.34 (4.58–6.23)
Stratified by gender			
Male	14129/1572620 (0.90)	4512/24882 (18.13)	24.43 (23.56–25.33)
Female	18996/2537316 (0.75)	6010/37798 (15.90)	25.06 (24.30–25.85)
Stratified by primary disease			
Schizophrenia	1060/27277 (3.89)	1398/4160 (33.61)	12.52 (11.45–13.68)
Bipolar disorder	649/23332 (2.78)	2459/10676 (23.03)	10.46 (9.56–11.45)
Stratified by drug combination			
Haloperidol	308/12393 (2.49)	1219/2712 (44.95)	32.04 (27.96–36.71)
Lithium	351/13116 (2.68)	785/3771 (20.82)	9.56 (8.38–10.91)

**Table 3:** Decrease of the occurrence of quetiapine-induced DM by a coexisting drug (drug B) in FAERS.

**Drug B**	**DM without drug B (%)**	**DM with drug B (%)**	**Odds ratio (95% CI)**
Pregabalin	10625/64076 (16.58)	184/1377 (13.36)	0.78 (0.66–0.91)
Lamotrigine	10387/61398 (16.92)	422/4055 (10.41)	0.57 (0.51–0.63)
Vitamin D	10743/64282 (16.71)	66/1171 (5.64)	0.30 (0.23–0.38)

The patient population was restricted to those receiving quetiapine. Top 3 coexisting drugs associated with decreased occurrence of DM-related adverse events are shown. The search terms for “vitamin D” are described in Supplementary Table S2.

**Table 4:** GEO data showing the effects of quetiapine on the expression levels of insulin resistance-related genes.

**Probe name**	**Gene symbol (Gene title)**	**Encoding protein**	**Relative expression**	***p*****-value**
NM_013005_PROBE1	*Pik3r1* (phosphatidylinositol 3-kinase, regulatory subunit, polypeptide 1)	PI3K	0.39 ± 0.13	0.0143
NM_013200_PROBE1	*Cpt1b* (carnitine palmitoyltransferase 1b, muscle)	CPT1β	0.46 ± 0.01	0.0186
L27112_PROBE1	*Mapk9* (mitogen-activated protein kinase 9)	JNK1	0.46 ± 0.08	0.0021
X95577_PROBE1	*Prkab1* (protein kinase, AMP-activated, beta 1 non-catalytic subunit)	AMPK	0.50 ± 0.12	0.0176
X73653_PROBE1	*Gsk3b* (glycogen synthase kinase 3 beta)	GSK3	0.63 ± 0.05	0.0025
L37085_PROBE1	*Frap1* (FK506 binding protein 12-rapamycin associated protein 1)	mTOR	0.66 ± 0.03	0.0001
NM_013089_PROBE1	*Gys2* (glycogen synthase 2)	GS	0.67 ± 0.06	0.0050
AI172465_PROBE1	*Ptpn11* (protein tyrosine phosphatase, non-receptor type 11)	PTPs	0.70 ± 0.07	0.0173
AF115282_PROBE1	*Ikbkb* (inhibitor of kappaB kinase beta)	IKKβ	0.70 ± 0.08	0.0396
Z29486_PROBE1	*Prkaa2* (protein kinase, AMP-activated, alpha 2 catalytic subunit)	AMPK	0.74 ± 0.05	0.0113
AI598719_PROBE1	*Mapk8* (mitogen-activated protein kinase 8)	JNK1	1.30 ± 0.09	0.0489

Expression levels are given as means ± SEM relative to those of the vehicle (carboxymethylcellulose)-treated group. *n* = 20–23 for vehicle; *n* = 3 for quetiapine-treated group.
