# Supplementary material for: Prevention of antipsychotic-induced hyperglycaemia by vitamin D: a data mining prediction followed by experimental exploration of the molecular mechanism
Source: Sci Rep. 2016 May 20;6:26375. doi: 10.1038/srep26375 (PMC4873813; doi:10.1038/srep26375)
Supplement: Supplementary Information [file srep26375-s1.doc]

**Supplementary Information**

**Prevention of antipsychotic-induced hyperglycaemia by vitamin D:
a data mining prediction followed by experimental exploration of the molecular mechanism**

**Takuya Nagashima^1^, Hisashi Shirakawa^1^, Takayuki Nakagawa^2^, Shuji Kaneko^1,*^**

^1^Department of Molecular Pharmacology, Graduate School of Pharmaceutical Sciences, Kyoto University, 46-29 Yoshida-Shimoadachi-cho, Sakyo-ku, Kyoto 606-8501, Japan

^2^Department of Clinical Pharmacology and Therapeutics, Kyoto University Hospital, 54 Shogoin-Kawahara-cho, Sakyo-ku, Kyoto 606-8507, Japan

***Corresponding author:**

Shuji Kaneko, Ph.D.

Department of Molecular Pharmacology,
Graduate School of Pharmaceutical Sciences, Kyoto University

46-29 Yoshida-Shimoadachi-cho, Sakyo-ku, Kyoto 606-8501, Japan

Tel: +81-75-753-4541, Fax: +81-75-753-4542

E-mail: skaneko@pharm.kyoto-u.ac.jp

**Supplementary Table S1.
Search terms for “DM” in the analysis of FAERS**

| Adverse event name | |
| --- | --- |
| Diabetes mellitus | Insulin-requiring type 2 diabetes mellitus |
| Type 2 diabetes mellitus | Diabetic hyperglycaemic coma |
| Diabetes mellitus inadequate control | Diabetic ketoacidotic hyperglycaemic coma |
| Diabetic ketoacidosis | Insulin-requiring type II diabetes mellitus |
| Diabetic coma | Diabetes with hyperosmolarity |
| Type 1 diabetes mellitus | Insulin resistant diabetes |
| Diabetes mellitus non-insulin-dependent | Latent autoimmune diabetes in adults |
| Diabetes mellitus insulin-dependent | Fulminant type 1 diabetes mellitus |
| Diabetic hyperosmolar coma |  |

| Resolved drug name | |
| --- | --- |
| Vitamin D | Calcifediol |
| Vitamin D-calcium combination | Maxacalcitol |
| Cholecalciferol | Eldecalcitol |
| Paricalcitol | Tacalcitol |
| 1-Hydroxycholecalciferol | Falecalcitriol |
| Calcitriol | Cholecalciferol-calcium combination |
| Ergocalciferol | Dihydroxycholecalciferol |
| Doxercalciferol |  |

**Supplementary Table S2.
Search terms for “vitamin D” in the analysis of FAERS**

**Supplementary Fig. S1.
Body weight was not affected by a vitamin D/cholecalciferol-supplemented diet.** Mice were fed a control or cholecalciferol-supplemented diet (1200 IU cholecalciferol/day) for 1 week and then fasted for 16 h prior to an intraperitoneal glucose administration. Body weights were measured daily during the experimental period. Data are given as means ± SEM (*n* = 14).

**Supplementary Fig. S2.
The anti-psychostimulant effect of quetiapine was not attenuated by vitamin D/cholecalciferol supplementation.** To determine whether vitamin D affects the therapeutic actions of quetiapine, we assessed the ability of cholecalciferol to affect the inhibitory effect of quetiapine on methamphetamine-induced hyperlocomotion, a representative preclinical marker of the positive symptoms of schizophrenia^1^. Mice were fed a control or cholecalciferol-supplemented diet for 1 week, followed by intraperitoneal administration of vehicle (distilled water plus 1% Tween 80), methamphetamine (2 mg/kg) or methamphetamine (2 mg/kg) plus quetiapine (10 mg/kg) at 30 min before locomotor activity test. Total distances travelled in the open-field chamber (50 cm × 50 cm × 50 cm) were measured during a 30-min test session using Any-maze Software (Stoelting Co., Wood Dale, IL, USA). Statistical analysis was performed using one-way ANOVA with post-hoc Tukey’s test. Data are given as means ± SEM (*n* = 4; *** *p* < 0.001 vs. vehicle-treated group (white column); # *p* < 0.05, ## *p* < 0.01 vs. methamphetamine-treated group (black column); N.S., not significant).

**Supplemental reference**

1. Kondo, M. A. et al. Unique pharmacological actions of atypical neuroleptic quetiapine: possible role in cell cycle/fate control. *Transl. Psychiatry* **3**, e243 (2013).
